# Feature-based attentional modulation of orientation perception in somatosensation

**DOI:** 10.3389/fnhum.2014.00519

**Published:** 2014-07-14

**Authors:** Meike A. Schweisfurth, Renate Schweizer, Stefan Treue

**Affiliations:** ^1^Cognitive Neuroscience Laboratory, German Primate CenterGoettingen, Germany; ^2^Biomedizinische NMR Forschungs GmbH am Max-Planck-Institut für biophysikalische ChemieGoettingen, Germany; ^3^Faculty for Biology and Psychology, Goettingen UniversityGoettingen, Germany; ^4^Bernstein Center for Computational NeuroscienceGoettingen, Germany

**Keywords:** attention, behavior, feature-based, human, orientation, reaction time, spatial, tactile

## Abstract

In a reaction time study of human tactile orientation detection the effects of spatial attention and feature-based attention were investigated. Subjects had to give speeded responses to target orientations (parallel and orthogonal to the finger axis) in a random stream of oblique tactile distractor orientations presented to their index and ring fingers. Before each block of trials, subjects received a tactile cue at one finger. By manipulating the validity of this cue with respect to its location and orientation (feature), we provided an incentive to subjects to attend spatially to the cued location and only there to the cued orientation. Subjects showed quicker responses to parallel compared to orthogonal targets, pointing to an orientation anisotropy in sensory processing. Also, faster reaction times (RTs) were observed in location-matched trials, i.e., when targets appeared on the cued finger, representing a perceptual benefit of spatial attention. Most importantly, RTs were shorter to orientations matching the cue, both at the cued and at the uncued location, documenting a global enhancement of tactile sensation by feature-based attention. This is the first report of a perceptual benefit of feature-based attention outside the spatial focus of attention in somatosensory perception. The similarity to effects of feature-based attention in visual perception supports the notion of matching attentional mechanisms across sensory domains.

## Introduction

Due to the brain’s limited processing capacity, human perception cannot provide a complete representation of the sensory input from the environment. Instead, our brain combines this external, bottom-up sensory information with internal, top-down influences to selectively enhance the processing and perception of information that we assume to be relevant. Voluntary attention is the major top-down influence for this selection process. It can lead to improved processing of attended locations, objects, and features, such as decreased reaction times (RTs) and higher accuracy rates for attended compared to unattended sensory signals (e.g., Posner, 1978). Perceptually, attention seems to enhance the integrated saliency (Treue, 2003) such as the perceived contrast and size of stimuli (Carrasco et al., 2004; Anton-Erxleben et al., 2007). While attentional effects have been extensively studied in the visual domain, far less research has been devoted to somatosensory attention (for an overview, see Mueller and Giabbiconi, 2008).

In touch, as in vision, the best-explored attentional phenomenon is spatial attention. Psychophysically, most research employed Posner (1978) or Posner-like designs. In these, a target has to be detected at one out of several possible locations. The target presentation is preceded by a cue indicating the likely target location; targets presented at that location are called validly cued, in contrast to invalidly-cued targets that are presented at another location. Using a Posner design with a simple detection task (same response button for all targets), some studies reported spatial-orienting effects (Butter et al., 1989; Cohen et al., 2005) whereas others did not find them (Posner, 1978) or only partly (presence vs. absence tasks in Sathian and Burton, 1991 and Whang et al., 1991; Lloyd et al., 1999). In tactile discrimination tasks (different response buttons for different targets) subjects often show faster reactions to validly-compared to invalidly-cued targets (Posner, 1978; Spence et al., 2000; Forster and Eimer, 2005; Chica et al., 2007; van Ede et al., 2012) or higher accuracy for validly-cued targets (Sathian and Burton, 1991; van Ede et al., 2012).

Whereas almost all attentional studies in touch have been focused on spatial attention, studies in the visual domain have shown that attention cannot only be allocated to specific regions of visual space but also to specific features. Here, “feature” refers to a particular value within a stimulus dimension. For example, upwards motion is a feature within the stimulus dimension of motion direction and red is a feature within the stimulus dimension of color. Just like spatial attention, feature-based attention can be demonstrated on the level of single neurons in sensory cortex. If a monkey’s attention is directed to the preferred feature (e.g., a color, a direction of motion) of an individual neuron, even far outside its receptive field, the neuron’s response will be increased (compared to a baseline where no feature is attended), whereas attention to the neuron’s non-preferred feature results in a decreased response (Treue and Martínez-Trujillo, 1999). This global effect of visual feature-based attention has also been shown in human psychophysical studies (Rossi and Paradiso, 1995; Sàenz et al., 2003), suggesting a higher accuracy for matching features. Human imaging studies (Saenz et al., 2002; Stoppel et al., 2011) extend these observations, reporting an increased fMRI response to an ignored stimulus of a given feature upon attention to a distant stimulus with the same feature compared to one with a different feature (Saenz et al., 2002). The feature-similarity gain model (Treue and Martínez-Trujillo, 1999) proposes a unified account for spatial and feature-based attentional modulation.

In the tactile domain, a couple of previous studies have employed tasks that required the perception of somatosensory features and found no evidence for improved performance in detecting sudden changes in those features by spatial attention (Sathian and Burton, 1991; Whang et al., 1991). Our study provides the first behavioral evidence for perceptual benefits of feature-based attention. We explored the behavioral effects of tactile spatial and feature-based attention in a human reaction time paradigm, using orientation as the relevant stimulus dimension. The task of the subjects was to monitor a stream of tactile stimuli for the occurrence of one of two designated target orientations. A cue specified the likely location and orientation of the target stimulus. As the orientation cue was only informative for the cued location, subjects were asked to attend to the cued location and–only there–the cued orientation. We observed faster RTs to orientation-matched compared to orientation-unmatched targets, both at the cued and the uncued location, indicating a global effect of feature-based attention in the human somatosensory system.

## Materials and methods

Twenty subjects (aged 24.8 ± 3.3 years (mean ± standard deviation), 11 males and 9 females) participated in this study. All subjects were right-handed (Edinburgh Inventory: laterality index 0.9 ± 0.1, Oldfield, 1971). They gave their informed written consent before the experiment. The study was approved by the ethics committee of the Georg-Elias-Mueller-Institute for Psychology, Goettingen University.

Each subject participated in three sessions of 2–3 h duration. The first session served as training, whereas the data recorded in the second and third sessions were used for analysis. Each session took place in a dimly illuminated and quiet testing room. Subjects sat on a comfortable chair, with their right foot placed on a foot pedal, such that it could be pressed by a small and effortless forefoot movement. The subjects’ hands were placed on a table, centrally in front of the body. After stimulator positioning (described below), the hands were covered by a sound-absorbing box, that did not touch the hands but ensured that tactile stimulation patterns could not be differentiated by visual or acoustic information. Subjects were told to keep their eyes closed throughout a session.

### Stimuli

**Figure 1:**
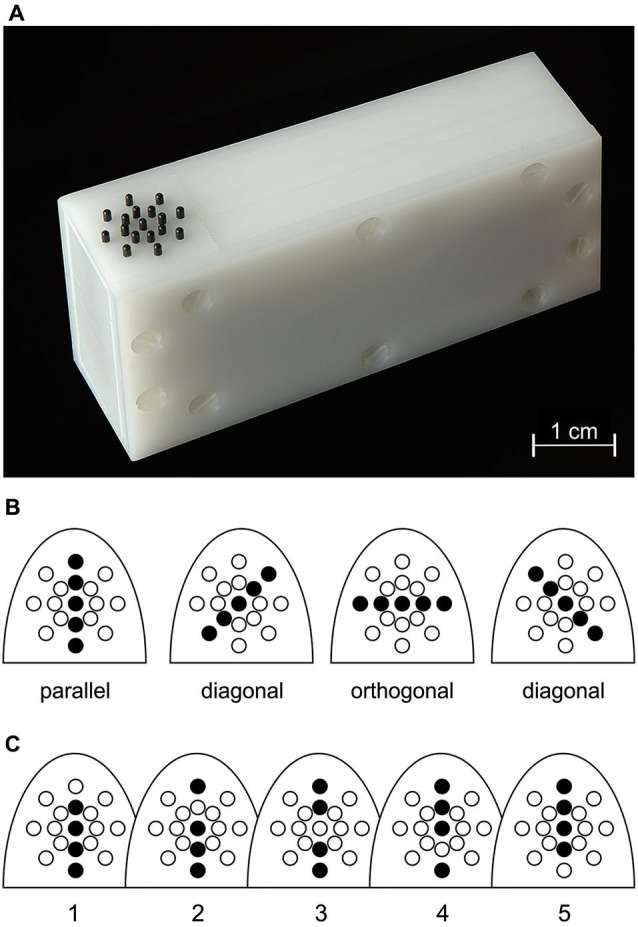
**Stimulation. (A)** Tactile stimulator, with the 17 radially-arranged black pins visible on the left. **(B)** Illustration of the four possible orientation patterns and their location relative to the proximal-to-distal fingertip axis.** (C)** Alternativ pin patterns used for a given orientation, exemplified here for the orientation parallel to the finger axis.

Tactile stimulation of the right-hand index (D2) and ring (D4) finger was performed using a piezo-electric stimulation device (Piezostimulator, QuaeroSys, St. Johann, Germany) consisting of a control unit and two connected, custom-built stimulation modules. Each module was equipped with a 17-pin radial display (Figure 1A) consisting of one central pin surrounded by two 8-pin circles of radius 2.5 and 5.0 mm, respectively. Each pin could be controlled individually.

By simultaneously elevating up to 5 pins arranged on a straight line through the central pin, 4 different orientations could be presented (Figure 1B): parallel to the finger axis (0°), orthogonal to the finger axis (90°) and the diagonal orientations in between (45° and 135°). The stimulation displays were positioned below the fingertips such that the parallel pin orientation was oriented along the proximal-to-distal fingertip axis and the central pin was located slightly distal to the fingertip vortex. Subjects were instructed to keep their right hand relaxed and pronated throughout the experiment. Each orientation presentation lasted for 1 s and consisted of 10 pin-raising cycles (stimulus duration = 50 ms, inter-stimulus interval = 50 ms), resulting in a stimulation frequency of 10 Hz. Pins were set to maximum drive-out (1.5 mm, if no weight was applied onto them). Orientation stimuli were generated by raising only 4 pins randomly chosen from the 5 pins forming the given orientation (Figure 1C). This procedure was applied to prevent subjects from solely concentrating on individual pins for solving the task.

Stimulation and recording of responses were each controlled using Apple Macintosh computers running in-house real-time stimulation and data-acquisition software.

### Design and procedure

**Figure 2:**
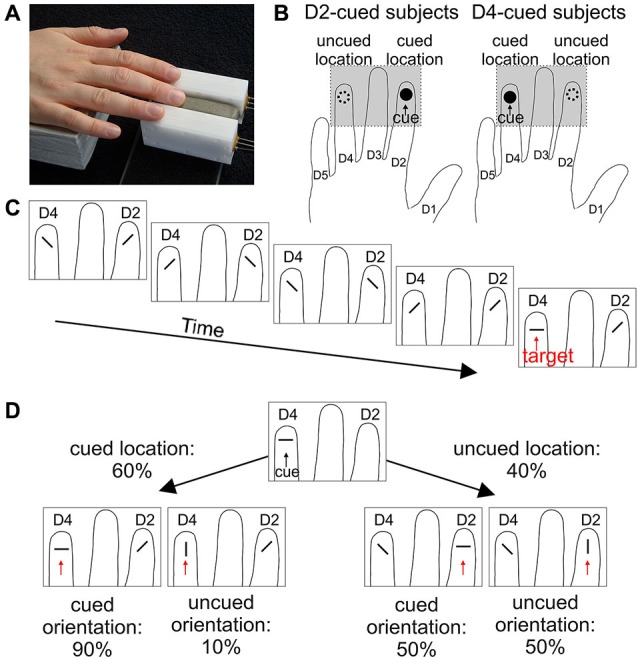
**Stimulus placement and paradigm. (A)** Placement of the right-hand index (D2) and ring (D4) finger onto the two radial stimulators shown in Figure 1. **(B)** Cue location. For both cue-location groups, the respective cued location (black circle), and the uncued location (dashed circle) are illustrated on a schematic hand. The gray rectangle defines the part shown in **C** and **D**. **(C)** Example of the sequence of events in a trial. Within each trial, subjects had to attend to the presentation of a random series of two diagonal orientations and react upon presentation of a parallel or orthogonal orientation (target) at any of the two locations. **(D)** At the beginning of each block, a cue with either orthogonal (shown here for a D4-cued subject) or parallel orientation was presented. Targets were more likely (60%) to occur at that cued location (panel in lower left) compared to the uncued location (40%, panel lower right). The orientation of the cue was only informative at the cued location, such that 90% of the targets presented there matched the orientation of the cue (orientation-matched targets); at the uncued location, both target orientations appeared with the same probability. Hence, in most of the trials (54%), targets matched the location and orientation of the cue. In the remaining trials, targets matched the location, but not the orientation of the cue (6% of all trials), or were presented at the uncued location with either matched or unmatched orientation (20% each).

Per session, 40 blocks of 10 trials each were acquired. The first trial of each block started 2 s after cue presentation. Trials within a block were separated by an inter-trial time of 1 s. The paradigm is illustrated in Figure 2.

In each trial (Figure 2C), independent random sequences of distractor orientations (oblique) were presented simultaneously at the right-hand index (D2) and ring (D4) finger (Figure 2A). The number of distractor presentations (3–15, mean of 6) was gamma distributed ~Γ(7.5, 0.8). Distractor stimuli were separated by 100 ms. At some point, a stimulus parallel or orthogonal to the finger axis (the “target”) was presented at one of the locations. The subjects were instructed to give a speeded response by pressing the foot pedal. Upon response (if within the 1 s stimulus interval) or 100 ms after target presentation, a mask stimulus was presented at both locations for 1 s, generated by repeated presentation of every second pin of the 17 pins.

In order to guide attention, each block started with a tactile cue (3 s duration), which was always presented at the same location. Half of the subjects received the cue at D2, the other half at D4 (Figure 2B). The cue was location-informative, as the targets were displayed at the cued finger in 60% of the trials (Figure 2D). Targets at this finger were therefore called location-matched, whereas targets at the other finger (only 40%) were called location-unmatched. The cue was of either parallel or orthogonal orientation (generated by elevation of all 5 pins) and orientation informative for location-matched targets, as these had the same orientation as the cue in 90% of the trials; for location-unmatched targets, the cue was non-informative, with parallel and orthogonal targets being equally likely (Figure 2D). A target was referred to as orientation-matched/-unmatched, if its orientation agreed/disagreed with the cue orientation. Subjects were instructed to make use of the information provided by the cue (i.e., location and orientation).

After each block, subjects could choose whether to go on or take a break in order to be able to maintain their level of concentration and tactile sensitivity. They were instructed to take at least one break within 100 trials.

### Analysis

Only RTs between 250 and 1350 ms after target onset were used for analysis, as shorter RTs likely were responses to the previous distractor and longer ones responses to the mask. RTs of each subject were sorted into the different combinations of target location and target orientation. For each subject and for each target location separately, RTs from each session were normalized to the subject’s overall mean and standard deviation (SD) and pooled across the two recording sessions in the following way. First, the population mean and SD were calculated for each target location, both separately for each session and jointly for both sessions (resulting in grand mean and grand SD). Then, separately for each session, the RTs were transformed into *z*-scores (by subtraction of the session mean followed by division by the session SD). Finally, these *z*-scores were transformed into normalized RTs by multiplication with the grand SD followed by addition of the grand mean. These normalized RTs could then be pooled across the two sessions of a subject and were used for further analysis.

Statistical analysis of RTs between attentional conditions was performed in SPSS (version 16.0). A four-way mixed analysis of variance (ANOVA) was conducted with the across-subjects factor cue-location group (D2-cued/D4-cued subjects) and the three within-subject, target-property factors location validity (location matched/unmatched), target orientation (parallel/orthogonal), and orientation validity (orientation matched/unmatched). Significant two-way interactions were broken down by simple-effects analysis, i.e., by pooling RTs across all but the two interacting factors and then calculating *post-hoc* paired *t*-tests between two levels of one factor, separately for the two levels of the other factor. Cohen’s *d* (Cohen, 1988; Erdfelder et al., 1996) was used as a measure of effect size.

## Results

The focus of our study was the effect of spatial and feature-based attention on behavioral performance in human somatosensation. After cueing one of two locations (either index or ring finger) and one of two orientations (either parallel or orthogonal, cue only informative for target presentations at the cued location) at the beginning of each block of trials, the subjects had to monitor two simultaneous sequences of oblique tactile distractors presented to the two fingers and react as soon as a target orientation (either parallel or orthogonal) was presented at one of the fingers (Figure 2). Subjects were instructed to attend to the cued location and orientation throughout a trial, as targets were more likely to appear at the cued location and with the cued feature (orientation). Comparing RTs to different combinations of target location and orientation, we assessed the effects of tactile spatial and feature-based attention.

Of the 800 trials performed per subject, 7.6 ± 3.0% (mean ± standard deviation) were excluded as early responses and 8.1 ± 4.6% as late responses (or because no response was given at all). Thus, the average success rate for the task (i.e., the percentage in which subjects responded in the accepted time window) across the 20 subjects was 84.3 ± 5.6%. On average, 674 ± 45 trials could be used per subject for further analysis.

**Figure 3:**
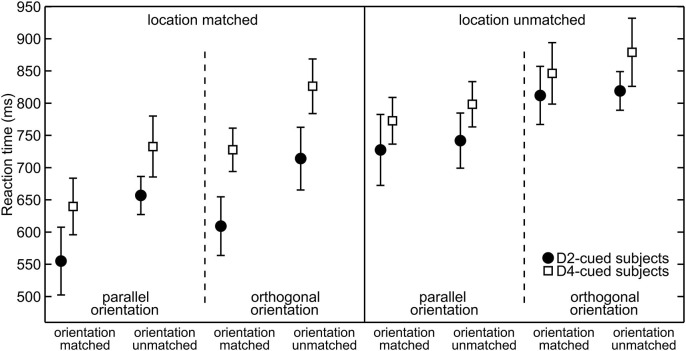
**Reaction times grouped with respect to target properties**. Separately for each cue-location group (for D2-cued/D4-cued subjects depicted by filled circles/open squares), the mean reaction times (RTs) (mean and repeated-measures 95% confidence intervals) are plotted. Responses to location-matched/location-unmatched targets are shown in the left/right half of the plot. Further on, these halfs are divided into parallel (left) and orthogonal (right) targets. The abscissa further sorts between orientation-matched (left) and orientation-unmatched targets (right).

To visualize spatial attention effects Figure 3 plots the average RTs. The data are grouped by the validity of the location cue (location-matched vs. location-unmatched) and within each validity condition grouped by the orientation of the target (parallel vs. orthogonal) to visualize differences in overall orientation sensitivity. For any given target orientation the RTs are separated by the validity of the orientation cue (orientation-matched vs. orientation-unmatched) to visualize effects of feature-based attention. The statistical significance of RT differences between conditions was evaluated with an overall four-way mixed ANOVA. The average RTs as a function of the four factors of interest (cue-location group, location validity, target orientation, and orientation validity) are listed in Table 1, the various effects are described below.

**Table 1:** **Reaction times grouped with respect to target properties**.

**Location validity**	**+**	**Target orientation**	**+**	**Orientation validity**	**D2-cued subjects M ± SD [ms]**	**D4-cued subjects M ± SD [ms]**
matched	+	parallel	+	matched	555 ± 70	640 ± 58
matched	+	parallel	+	unmatched	657 ± 39	733 ± 63
matched	+	orthogonal	+	matched	609 ± 60	728 ± 45
matched	+	orthogonal	+	unmatched	714 ± 65	826 ± 56
unmatched	+	parallel	+	matched	728 ± 73	773 ± 48
unmatched	+	parallel	+	unmatched	742 ± 57	798 ± 47
unmatched	+	orthogonal	+	matched	812 ± 60	846 ± 63
unmatched	+	orthogonal	+	unmatched	819 ± 40	879 ± 70

*Separately for each cue-location goup, the reaction times (mean ± repeated-measures standard deviation) in response to targets are listed for each set of target properties, i.e., for each combination of location validity, target orientation, and orientation validity*.

### Spatial attention

**Figure 4:**
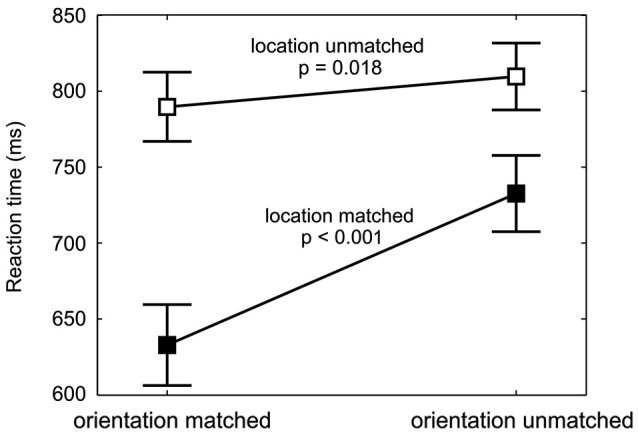
**Spatial and feature-based cueing benefit**. RTs (mean and repeated-measures 95% confidence intervals, pooled across cue-location groups and target orientations) are shown for each combination of location validity and orientation validity. The values for targets with different orientation validities but same location validity are connected (filled data points for location-matched, empty data points for location-unmatched targets). At both, the cued and at the uncued location, a significant decrease in reaction time from orientation-unmatched to orientation-matched targets is found, with a larger effect for location-matched targets.

At the cued location, subjects reacted on average 117 ms faster than at the uncued location. This effect is visible in Figure 3, where RTs under identical sensory conditions are lower in the left (“location-matched”) compared to the right half (“location-unmatched”) of the plot. The effect is also visible in Figure 4. Statistically, it was confirmed by a significant main effect of location validity in the ANOVA (mean difference (*M*) = −117 ms, repeated-measures standard deviation (*SD*) = 89 ms, *F*_(1,18)_ = 35.6, *p* < 0.001). Hence, spatial allocation of attention resulted in decreased RTs at the cued location.

### Feature-based attention

RTs to targets with the cued orientation were on average 60 ms faster than orientation-unmatched targets. This is apparent in Figure 3, as under otherwise identical conditions (neighboring same-marker data points without line separation) RTs to orientation-matched targets (left two values in each group of four) are lower than those to orientation-unmatched targets (right two values in each group of four). This observation was statistically confirmed by a significant main effect of orientation validity (*M* = −60 ms, *SD* = 46 ms, *F*_(1,18)_ = 32.8, *p* < 0.001).

The feature-based decrease in RTs was strong at the cued location, where responses to the cued orientation were 99 ms faster, but also detectable at the uncued location, where the RT difference amounted to 20 ms. Figure 4 shows this increase in RT between orientation-matched and orientation-unmatched targets both for location-matched (large increase) and for location-unmatched targets (small increase). Statistically, this was reflected in a significant interaction between the factors location validity and orientation validity (*F*_(1,18)_ = 48.1, *p* < 0.001). Follow-up simple-effects analysis confirmed that RTs to the cued orientation were significantly faster both at the cued and at the uncued location. While the effect was large for location-matched targets (*M* = −99 ms, *SD* = 66 ms, *t*_(19)_ = −6.79, *p* < 0.001, effect size *d* = 1.5), it was of medium effect size for location-unmatched targets (*M* = −20 ms, *SD* = 23 ms, *t*_(19)_ = −2.59, *p* = 0.018, *d* = 0.6). Hence, feature-based allocation of attention not only has an influence at the location where the feature-based cue is informative, but also at the location without any previous feature-based information, documenting a global effect of feature-based attention.

### Orientation anisotropy

**Figure 5:**
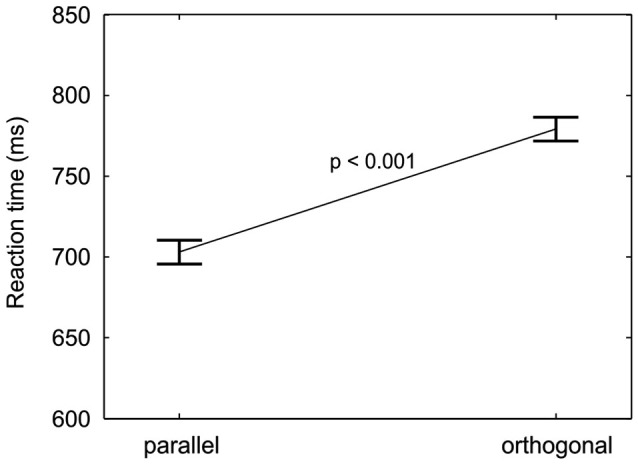
**Orientation anisotropy**. RTs (mean and repeated-measures 95% confidence intervals, pooled across cue-location groups, location validities, and orientation validities) are plotted for the two target orientations. A significant decrease in reaction time from orthogonal to parallel targets is found.

Responses to targets parallel to the finger axis were on average 76 ms faster than responses to orthogonal targets, as can be seen in Figure 5. The effect was statistically confirmed by a significant main effect of target orientation (*M* = −76 ms, *SD* = 47 ms, *F*_(1,18)_ = 51.9, *p* < 0.001). The RT difference between targets with parallel and orthogonal orientation points to an anisotropy in orientation processing or perception.

### Further results

Subjects for whom the index finger (D2) formed the cued location tended to respond faster (729 ± 30 ms) than D4-cued subjects (753 ± 23 ms). That trend is reflected in Figure 3, where the black marker tends to be lower than its adjacent white marker. However, the trend did not reach statistical significance, as seen by the main effect of cue-location group (*M* = −73 ms, *F*_(1,18)_ = 3.5, *p* = 0.079).

All interactions between within-subject factors were far from significant (*F*_(1,18)_ < 1, *p* = 0.7), except for the already discussed interaction between location validity and orientation validity. Also, all interactions of the across-subject factor cue-location group with one, two, or three of the within-subject factors proved insignificant (*F*_(1,18)_ < 1.5, *p* = 0.2).

## Discussion

To determine the presence and document the consequences of spatial and feature-based attention in the somatosensory system, we studied such influences on human RTs. Subjects had to report the presentation of target orientations (parallel and orthogonal) to their index and ring fingers, ignoring oblique distractor orientations in a rapid serial presentation. Our data show faster RTs not only at the attended finger (i.e., the location of spatial attention), but also globally for the attended feature. Additionally, responses were faster for parallelly compared to orthogonally oriented stimuli.

### Spatial attention

Our data show that responses to targets at the cued location are much faster compared to those at the uncued location, in line with several previous studies (Posner, 1978; Spence et al., 2000; Chica et al., 2007) reporting a behavioral RT effect of spatial cueing in touch.

In many other psychophysical studies, Posner-like RT paradigms have been used and whenever RTs decreased for validly- compared to invalidly-cued features or locations, this is typically attributed to an attentional acceleration of the processing of information from the attended location. However, improved perceptual performance is not necessarily evidence for more efficient cortical processing (a consequence of attentional selection; Duncan, 1980; Sperling, 1984). Decreased RTs as a function of the information by the cue can also result from lowering the amount of sensory information required for triggering a response, i.e., by decreasing the level of required certainty. As a target at the cued location was more likely (60%) than at the uncued location, the effect of faster RTs at the cued location might, at least partly, result from a higher expectancy or a higher likelihood of location-matched compared to location-unmatched targets.

### Feature-based attention

Using a psychophysical paradigm designed to investigate global effects of feature-based attention our data show perceptual benefits when the subjects’ attention was directed to behaviorally-relevant tactile features. Responses are faster for orientation-matched targets at the location for which the feature-based cue was orientation-informative (cued location). However, similar to the spatial attention effects described above, it is unclear whether these effects are due to faster processing resulting from allocation of feature-based attention or due to lowering the amount of information required for triggering a response resulting from the higher probability for a matched target (90% vs. 10% at the cued location).

Crucially, however, decreased RTs were not only observed at the cued location but also at the uncued location, documenting a global effect of tactile feature-based attention. At the uncued location, different degrees of certainty between valid and invalid targets cannot account for the effect, as both conditions occurred equally often.

The global effect of feature-based attention we observed is similar to the one reported by psychophysical studies in vision (Rossi and Paradiso, 1995; Alais and Blake, 1999; Sàenz et al., 2003; Arman et al., 2006) and represents the first report of behavioral effects of feature-based attention in touch. Thereby, it extends prior observations (Sathian and Burton, 1991; Whang et al., 1991) showing evidence for feature detectors for grating and intensity changes in the tactile modality. Our findings are also well complemented by the only other study on tactile feature-based attention (Forster and Eimer, 2004), which reported cortical evidence for global effects. In their study, event-related potentials (ERPs) were recorded upon delivery of tactile stimuli presented to the right or left hand. Stimuli were of low or high frequency (first experiment) or of low or high intensity (second experiment). Subjects had to attend simultaneously to one of the stimulus locations and to one of the non-spatial features. ERP analysis revealed effects of feature-based attention (enhanced negativities to the attended frequency or intensity), independent of the current focus of spatial attention, suggesting a global effect of feature-based attention. Perceptual effects were not assessed in the cited study. Further imaging studies will be necessary to identify the cortical regions in which tactile feature-based attention operates.

### Finger anisotropy

Across subjects we observed a trend for lower RTs to targets presented to D2 compared to targets at D4. This finding is in agreement with a study by Vega-Bermudez and Johnson (2001) who reported that tactile acuity in a letter-recognition and in a grating-orientation discrimination task progressively declined from D2 to D4, suggesting anisotropic sensitivities between the fingers, possibly because of the more important role of the index finger (compared to the ring finger) in everyday hand-use. Duncan and Boynton (2007) further reported that this effect was reflected in the primary somatosensory cortex (SI), as the increase in tactile threshold from D2 to D4 was correlated with a decrease in digit area from D2 to D4 in SI.

### Orientation anisotropy

There is an ongoing debate about anisotropic processing and perception of tactile orientations either aligned (“parallel” throughout this text) or orthogonal (“orthogonal” throughout here) to the finger axis. Lechelt (1988) reported better detection of deviations from orthogonal compared to parallel orientations. Similarly, Bensmaia et al. (2008b) observed a lower angular deviation-detection threshold for the orthogonal compared to the parallel orientation and a trend for better performance for orthogonal compared to bars parallel to the finger axis, both in an orientation-discrimination and in a convergence-detection task. Essock et al. (1997) reported that sensitivity to detection of gratings (vs. blanks) was best for gratings parallel to the finger axis and worst for orthogonal ones. The results could not be replicated by a similar study by Craig (1999), whereas Gibson and Craig (2005) found similar results for the finger location stimulated in our design (defined as fingerpad in their study). In a gap-detection and in a grating orientation (GR/OR) task, however, these authors could not find anisotropy between the parallel and the orthogonal orientation. In rhesus monkeys (DiCarlo and Johnson, 2000), “parallel” was reported less often as a neuron’s preferred orientation in the SI than other orientations (not statistically tested), whereas Bensmaia et al. (2008a) did not find any orientation to be overrepresented in SI. Alternatively, it has been suggested in a study on humans and monkeys that orientation parallel to the finger ridges is best detected (Wheat and Goodwin, 2000). However, in contrast to monkeys, the rigde pattern of fingerpads is not consistent across humans.

Our results show anisotropy between target orientations, as parallel targets led to significantly faster responses than orthogonal targets. Without being questioned, seven out of the 20 subjects stated that the detection of parallel targets was easier for them, while none claimed the contrary. Interpreting the results in light of previous studies, one could argue that faster responses to parallel targets resulted from worse detection of deviations from a parallel compared to those from an orthogonal standard orientation (Lechelt, 1988; Bensmaia et al., 2008b). Oblique-stimulus presentation before target appearance might further alter the subjects’ perception of parallel and orthogonal orientation. As even large deviations from the parallel orientation might still be categorized as “parallel”, parallel targets might have appeared clearer and hence were more quickly detectable. However, as we did not measure the exact amount of skin displacement for the parallel compared to the orthogonal orientation, the observed orientation anisotropy might also partly have resulted from differences in physical stimulus strength, potentially leading to a higher perceptual strength for the parallel orientation.

## Conclusion

In conclusion, this study not only confirms effects of tactile spatial attention but is the first to report behavioral effects of tactile feature-based attention, acting not only on a local but on a global scale, similar to the effects observed in vision. The presence of feature-based effects at the uncued location strongly supports that behavior is not (only) altered due to cue-related modification in expectancies but because of altered cortical information processing upon feature-based attention. Further cortical studies are needed to identify the brain areas in which the reported behavioral effects of feature-based attention originate.

## Conflict of interest statement

The authors declare that the research was conducted in the absence of any commercial or financial relationships that could be construed as a potential conflict of interest.
